# The journey not the destination: Liminality and lifelong learning

**DOI:** 10.1111/medu.14956

**Published:** 2022-10-27

**Authors:** Helen Jones, Lucy Hammond

**Affiliations:** ^1^ Warwick Medical School University of Warwick Coventry UK

## Abstract

If we are to treat adaptive expertise as a threshold concept, the authors argue we must consider it a journey rather than a destination, requiring learners to respond to novel situations in innovative ways.

*Thinking like* and *being* a practitioner requires more than knowledge and proficiency in discrete skills and is an ongoing learning and development process. In this issue of *Medical Education*, Betinol et al report on a study in which they interviewed recently graduated physical therapists to explore their understandings of expertise.^1^ The authors found that newly qualified physical therapists oscillate between conceptions that align with routine expertise and those that align with adaptive expertise. In considering their findings with wider bodies of work in medicine and the health professions, the authors suggest that adaptive expertise ‘may be best understood as a threshold concept’.^1^

A key theme of Betinol et al's study is the ongoing learning journey that graduates experience in the development of adaptive expertise and the need to better support this through continuing professional development programmes.^1^ During the study interviews, one participant came to the realisation that ‘expertise is a never‐ending process; it's not a fixed point’.^1^ Similarly, Kua et al described how ‘through the iterative process of problem solving and guided successful transfer experiences expertise can be continuously refined and expanded’.^2^ These observations are consistent with many of the TCs identified in our recent scoping review of the threshold concept framework (TCF) in medical education,^3^ which involve an ongoing series of ontological shifts and nuanced refinements in understandings as new, increasingly complex experiences and learning transformations are encountered through clinical practice. By considering TCs through the lens of lifelong learning, we conceptualise some TCs as recursive, located in an ongoing state of liminality; they are an *ongoing journey* rather than a *destination to be reached*. In this context, we find the singular nature of the TC metaphor—depicting the crossing of *a* portal or stepping through *a* doorway to new understanding and enlightenment—somewhat limiting.

We conceptualise some TCs as recursive, located in an ongoing state of liminality; they are an *ongoing journey* rather than a *destination to be reached*.

For learners, this recursivity may align with changing environments or responsibilities, requiring them to develop a more advanced understanding or embodiment of a TC.^3^ With adaptive expertise, this transition point appears to occur when graduates gained individual responsibility for patient care, and they demonstrated a developing awareness of adaptive perspectives.^1^ However, they did not yet appreciate the importance of balancing both routine (efficient) and adaptive (innovative) approaches in their practice. Being able to balance these two approaches in response to problems—the ‘optimal adaptability corridor’—is an essential characteristic of an adaptive expert.^4^ Adaptive expertise, therefore, may also be a recursive TC, in which there is at first understanding of innovative approaches and later competent utilisation of both efficiency and innovation. This involves a career‐long journey with repeated hurdles, not a single doorway that is traversed.

There is at first understanding of innovative approaches and later competent utilisation of both efficiency and innovation.

This perspective leads us to consider other TCs that may underpin adaptive expertise development. TCs have previously been described in context of web, in which acquisition of one TC further transforms understanding of another.^5^, ^6^ In exploring adaptive expertise, Betinol et al highlight that students ‘persistently attempt to impose certainty on inherently uncertain clinical situations’^7^ and contrast this desire for knowledge‐based, routine, predictable, standardised certainty with clinical expertise, which is characterised by adaptive problem solving, unpredictability, dynamic practice and uncertainty.^1^ If creative problem solving is required to address the uncertainty of complex clinical situations, then it follows that recognising, accepting and embodying uncertainty as a feature of clinical practice is *first* required. Uncertainty is a key TC found at all stages of the medical education continuum and underlies aspects of clinical reasoning and decision making.^3^ Therefore, a better understanding of connections between concepts in the health professions is needed to distinguish what concepts are strands, steps or TCs in themselves and how they influence one another. Concept mapping has been used in engineering to express the interrelationship and contingencies between the most integrative TCs, where integrative is taken to mean connecting all disciplines.^8^ This reveals clusters of TCs, overarching TCs and TCs of different levels of specificity and provides a sense of their relationships. Such an approach could enhance understanding of complex TCs in health care, as has been explored for nurturing care among junior doctors.^9^

A better understanding of connections between concepts in the health professions is needed.

Understanding how training can prepare learners for encountering recursive TCs such as adaptive expertise and uncertainty is of fundamental importance. The Threshold Concept Integrated Capability Framework (TCICF) could be helpful in this regard. It has been proposed that innovation in practice requires the development of capability, by learners adapting and applying competencies in new contexts.^10^ The TCITF combines the TCF with capability theory, creating a pedagogical approach for developing the capability of graduates to deal with unseen professional situations.^6^ The TCF forefronts episteme (knowledge of the concept; the ‘what’) and capability theory forefronts phronesis (judgement and decision making; the ‘how’) and in combination can aid the development of knowledge capability: the ability to assess a new situation, determine the problem and design a solution.^6^

This perspective could help identify educational practices to aid navigation through the ongoing liminal space. As with adaptive expertise, reflection is a key component of developing capability,^10^ but this needs to be accompanied by experience of variation.^6^ Only by exposure to new situations in which learners must decipher *what* knowledge is required to identify and solve the problem, followed by active reflection on *why* the situation and necessitated approach is different from those previously experienced, can learners develop the capability to deal with new and unforeseen circumstances.^6^ This is likely to require a range of learning methods, including problem‐based learning, case‐based discussions and feedback on performance.^10^ Furthermore, traditional assessments methods typically focus on routinisation, and work is required to develop approaches for successfully evaluating adaptive expertise.^1^ We feel that this should include assessment of capability and learners' skills in approaching new situations rooted in professional practice.

Reflection is a key component of developing capability, but this needs to be accompanied by experience of variation.

Adaptive expertise is an example of a troublesome TC that does not represent a sudden ‘aha moment’, but, like many of the TCs identified in medical education, represents an ongoing liminal journey through multiple transformations that accompany the development of professional practice. To be an adaptive expert, learners need to develop the capability to respond to novel situations in an innovative way. How we help students do this is one of the most important questions facing health professions education today.

To be an adaptive expert, learners need to develop the capability to respond to novel situations in an innovative way.

## References

[1] Betinol E , Murphy S , Regehr G . Exploring the development of adaptive expertise through the lens of threshold concepts. Med Educ. 2022;57(2):142‐50. doi:10.1111/medu.14887 35918846

[2] Kua J , Lim W , Teo W , Edwards RA . A scoping review of adaptive expertise in education. Med Teach. 2021;43(3):347‐355. doi:10.1080/0142159X.2020.1851020 33251895

[3] Jones H , Hammond L . Threshold concepts in medical education: a scoping review. Med Educ. 2022;56(10):983‐993. doi:10.1111/medu.14864 35775904 PMC9543879

[4] Mylopoulos M , Regehr G . How student models of expertise and innovation impact the development of adaptive expertise in medicine. Med Educ. 2009;43(2):127‐132. doi:10.1111/j.1365-2923.2008.03254.x 19161482

[5] Davies P , Mangan J . Threshold concepts and the integration of understanding in economics. Stud Higher Edu. 2007;32(6):711‐726. doi:10.1080/03075070701685148

[6] Baillie C , Bowden JB , Meyer JHF . Threshold capabilities: threshold concepts and knowledge capability linked through variation theory. High Educ. 2013;65(2):227‐246. doi:10.1007/s10734-012-9540-5

[7] White G , Williams S . The certainty of uncertainty: can we teach a constructive response? Med Educ. 2017;51(12):1200‐1202. doi:10.1111/medu.13466 29124799

[8] Male S . Engineering thresholds: an approach to curriculum renewal. 2012. https://ltr.edu.au/resources/PP10_1607_Baillie_Inventory_2012.pdf

[9] Wilkinson I . Nurturing and complexity – threshold concepts in geriatric medicine. Int J Pract‐Based Learn Health Social Care. 2018;6(1):64‐77. doi:10.18552/ijpblhsc.v6i1.420

[10] Fraser SW , Greenhalgh T . Coping with complexity: educating for capability. BMJ. 2001;323(7316):799‐803. doi:10.1136/bmj.323.7316.799 11588088 PMC1121342

